# Telomeres, A Busy Platform for Cell Signaling

**DOI:** 10.3389/fonc.2013.00146

**Published:** 2013-06-10

**Authors:** Laura Gardano, Fabio Pucci, Larissa Christian, Thierry Le Bihan, Lea Harrington

**Affiliations:** ^1^Wellcome Trust Centre for Cell Biology, University of Edinburgh, Edinburgh, UK; ^2^SynthSys Synthetic and Systems Biology, University of Edinburgh, Edinburgh, UK; ^3^Institut de Recherche en Immunologie et Cancérologie, Université de Montréal, Montréal, QC, Canada

**Keywords:** telomere, telomerase, shelterin, Wnt signaling, β-catenin, APC

## Abstract

Telomeres are the terminal structures at the ends of linear chromosomes that represent a solution to the end replication problem. Specific binding of the six-protein subunit complex shelterin to telomeric, repetitive TTAGGG DNA sequences contributes to the stable architecture and maintenance of telomeres. Proteins involved in the DNA damage response are also localized at telomeres, and play a role in the surveillance and maintenance of telomere integrity. The enzyme responsible for telomere extension is telomerase, a ribonucleoprotein with reverse transcriptase activity. In the absence of telomerase, telomeres shorten to a length threshold that triggers the DNA damage response and replicative senescence. Here, we will summarize the latest findings concerning vertebrate telomere structure and epigenetics, and we present data regarding the impact of short telomeres upon cell signaling. In particular, in murine embryonic stem cells lacking telomerase, we found that distribution of cytosolic/nuclear β-catenin, a key component of the Wnt signaling pathway, changes when telomeres become critically short. We discuss implications and future perspectives of the effect of epigenetic modifications and/or conformational changes of telomeres on cell metabolism and signaling networks. Such an analysis may unveil potential therapeutic targets for pathologies like cancer, where the integrity of telomeres is altered.

## Introduction

### Telomere structure in vertebrates

**Figure 1:**
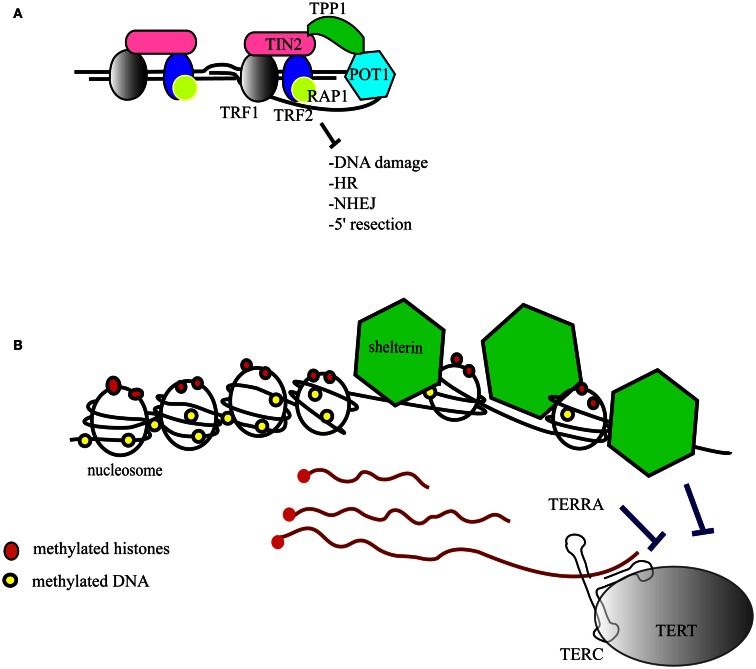
**Structure of the telomeres**. **(A)** The telomere folds into a telomeric loop (t-loop) and binds the six-protein subunit complex shelterin. TRF1 (in gray) and TRF2 (in blue) bind double-stranded telomeric DNA. POT1 (in light blue) binds to single-stranded telomeric DNA. RAP1 (in yellow) is recruited to the telomere by an interaction with TRF2. TIN2 (in pink) serves as a scaffold to recruit TRF1, TRF2, and TPP1 (in green), which in turn interacts with POT1. The main functions of shelterin are listed in the figure and further details are given in the text. HR, homologous recombination; NHEJ, non-homologous end joining. **(B)** Epigenetic marks at the telomere (red dots represent histone methylation and yellow dots represent DNA methylation). The shelterin complex (green) regulates nucleosome spacing. The telomeres are transcribed into TERRAs (red strings) that inhibit telomerase catalytic activity, and shelterin inhibits telomerase access. TERC, the RNA component of telomerase; TERT, the catalytic subunit of telomerase.

Telomeres are the structures at the ends of chromosomes that protect them from end-to-end fusions and solve the problem of end replication, i.e., the loss of genetic material due to inherent limitations in the DNA replication process (Blackburn, 1991). Telomeres consists of a repeated six-nucleotide G-rich sequence, 5′-TTAGGG-3′, that is folded into a telomeric loop (t-loop) (Griffith et al., 1999). The telomere contains a double-stranded region and a single-stranded overhang, also referred to as the G-strand overhang, whose length is tightly regulated (Wright et al., 1997; Sfeir et al., 2005; Wu et al., 2012). Telomeres are protected and regulated by a specific hexaprotein complex, called shelterin (i.e., TRF1, TRF2, RAP1, TIN2, POT1, TPP1) (Figure 1A), and additional non-telomere specific proteins that are implicated in the cellular DNA damage response (de Lange, 2005; Longhese, 2008). Shelterin inhibits the ataxia telangiectasia mutated (ATM) and ATM and Rad3-related (ATR)-dependent DNA damage response, non-homologous end joining and homologous recombination DNA repair pathways, and resection by 5′-exonucleases (Sfeir and de Lange, 2012). Some of these activities are specific to shelterin whereas other activities that inhibit non-homologous end joining and resection are supported by other telomere-associated proteins such as Ku70/80 and 53BP1, respectively (Sfeir and de Lange, 2012). As the enzyme responsible for telomere extension, telomerase is a key factor that contributes to chromosome end protection (Blackburn et al., 1989). Telomerase is a reverse transcriptase that copies a stably associated RNA template into telomere DNA (Greider and Blackburn, 1987, 1989; Shippen-Lentz and Blackburn, 1990). In mice, the extension of telomeres occurs during S-phase and telomerase extends the shortest telomeres preferentially (Hemann and Greider, 1999; Samper et al., 2001; Erdmann et al., 2004; Stern and Bryan, 2008).

### Telomere epigenetics

Telomeric DNA contains nucleosomes, although the nature of telomeric chromatin is peculiar (Makarov et al., 1993) (Figure 1B). Simplistically, nucleosomes and shelterin compete with each other for the binding of telomeric DNA, hence it is not surprising that TRF2 influences the positioning of the nucleosomes, i.e., the nucleosome abundance at telomeres is inversely correlated with the amount of TRF2 (Benetti et al., 2008b; Galati et al., 2012). Nucleosome spacing by TRF2 occurs in S/G2 phase, which coincides with the end of DNA replication and telomere replication (Galati et al., 2012). Epigenetic marks, notably histone and DNA methylation, in sub-telomeric and telomeric regions contribute further to telomere maintenance and stability (Blasco, 2007) (Figure 1B). A high degree of DNA methylation guarantees a closed chromatin state that is associated with gene silencing in regions upstream of the telomeres. This phenomenon, first described in *Drosophila melanogaster* and yeast, is known as the telomere positioning effect or TPE (Levis et al., 1985; Gottschling et al., 1990; Nimmo et al., 1994). In humans, telomere length positively affects TPE through a change in the conformation of chromatin (Baur et al., 2001). Epigenetic defects at telomeres, such as those driven by the loss of DNA methyl transferases or histone methyl transferases, lead to telomere defects that result in aberrant telomere lengthening attributed partially to an increase in homologous recombination (Gonzalo et al., 2006; Benetti et al., 2007b, 2008a). In mice lacking the telomerase RNA component (*mTerc^−*/*−^*), short telomeres are associated with epigenetic changes at the telomeres, i.e., a decrease of tri-methylated histone 3 and histone 4 and an increase in histone acetylation (Benetti et al., 2007a). Thus, critically shortened telomeres show signs of an “open” chromatin state that favors recombination events (Benetti et al., 2008a). It is reasonable to postulate that epigenetic changes in telomeric DNA and histones affect the binding of shelterin and, in turn, affect telomere structure and the recruitment of telomerase (Blasco, 2007).

The complex regulation of telomeric structure, maintenance, and epigenetics has been underscored further by the discovery of the transcription of telomeres into a telomere repeat-containing RNA (TERRA) that contains UUAGGG repeats (Azzalin et al., 2007; Schoeftner and Blasco, 2008) (Figure 1B). The length and amount of TERRAs are directly correlated with telomere length and vary with the cell cycle. Because of their ability to anneal with the template sequence in the telomerase RNA component, TERRAs are able to inhibit telomerase (Figure 1B) (Redon et al., 2010). The precise role of TERRAs has not yet been established fully, but TERRAs are proving to be an interesting regulator of telomere dynamics.

### Telomeres, telomerase, and the Wnt signaling pathway

Telomere dysfunction is also linked to perturbation of other cellular processes that include the Wnt/β-catenin signaling network. The Wnt/β-catenin signaling cascade controls many aspects of organism development, cell proliferation, and differentiation (Valenta et al., 2012). In the absence of Wnt, β-catenin is phosphorylated and rapidly degraded by a destruction complex containing Axin, APC, CK1, and GSK3β (Clevers and Nusse, 2012). However, in the presence of Wnt, β-catenin is stabilized and imported into the nucleus where, together with the transcription complex TCF/LEF, it regulates the transcription of Wnt target genes (Behrens et al., 1996; Molenaar et al., 1996). Cytoplasmic β-catenin localizes to the cell membrane through an interaction with E-cadherin and serves to stabilize cell adhesion (Ozawa et al., 1989).

The first link between telomerase and Wnt signaling was suggested from an analysis of transcription profiles of mouse and human cells expressing catalytically active or inactive Tert (Choi et al., 2008). Stem cells that express mTert, irrespective of its competence for catalytic activity, exhibit transcriptional activation of genes regulated by Wnt (Choi et al., 2008). In addition, mTert is localized to the promoters of genes regulated by Wnt3a and β-catenin (Park et al., 2009). In mESC over-expressing mTert, the activation of Wnt signaling by LiCl leads to the transcriptional activation of β-catenin (Park et al., 2009). However, another study compared the transcriptional profile of cells from *mTert^−*/*−^* mice with *mTerc^−*/*−^* mice, and observed no substantial difference in gene expression (Vidal-Cardenas and Greider, 2010). In particular, the Wnt signaling network was unaffected, and the authors suggested that the link between telomerase and Wnt signaling might be a neomorph due to telomerase over-expression (Strong et al., 2011). More recently, it has been found that β-catenin can regulate *mTert* transcription in mESC (Hoffmeyer et al., 2012). This regulation involves Klf4, one of the four transcription factors required to induce pluripotent stem cells. The control operated on *mTert* by β-catenin may be direct because β-catenin occupies the *mTert* promoter (Hoffmeyer et al., 2012). β-catenin also activates *TRF2* transcription (Diala et al., 2013). Finally, *c-myc*, which is also under the control of β-catenin/Wnt signaling, is a known regulator of *mTert* transcription (Wang et al., 1998), thus implying a very tight regulation of this gene and the involvement of multiple signaling networks (Greider, 2012).

Telomere attrition triggers activation of the DNA damage response and other changes that herald the onset of genome instability (Cimprich and Cortez, 2008; Schoeftner and Blasco, 2010). To dissect the complexity of such processes, it is important to distinguish between the impact of telomerase loss versus the impact on telomere length. In this regard, murine embryonic stem cells represent a valuable model system and in ESC lacking *mTert*, we show that critically short telomeres (and not telomerase presence *per se*) can impact cell signaling cascades even in the cytoplasm. We focused on β-catenin because of its known link to telomere function and because its dynamic phosphorylation-dependent regulation appeared a logical choice for a first examination of the impact of DNA damage signaling at the telomere in the cytoplasm. Our data suggest that alteration of telomere structure or epigenetic modifications elicited by telomere shortening impacts cell signaling in extra-nuclear locations, which in turn may affect cell adhesion, metabolism, and protein turnover.

## Results

### Short telomeres affect cell adhesion and β-catenin distribution

**Figure 2:**
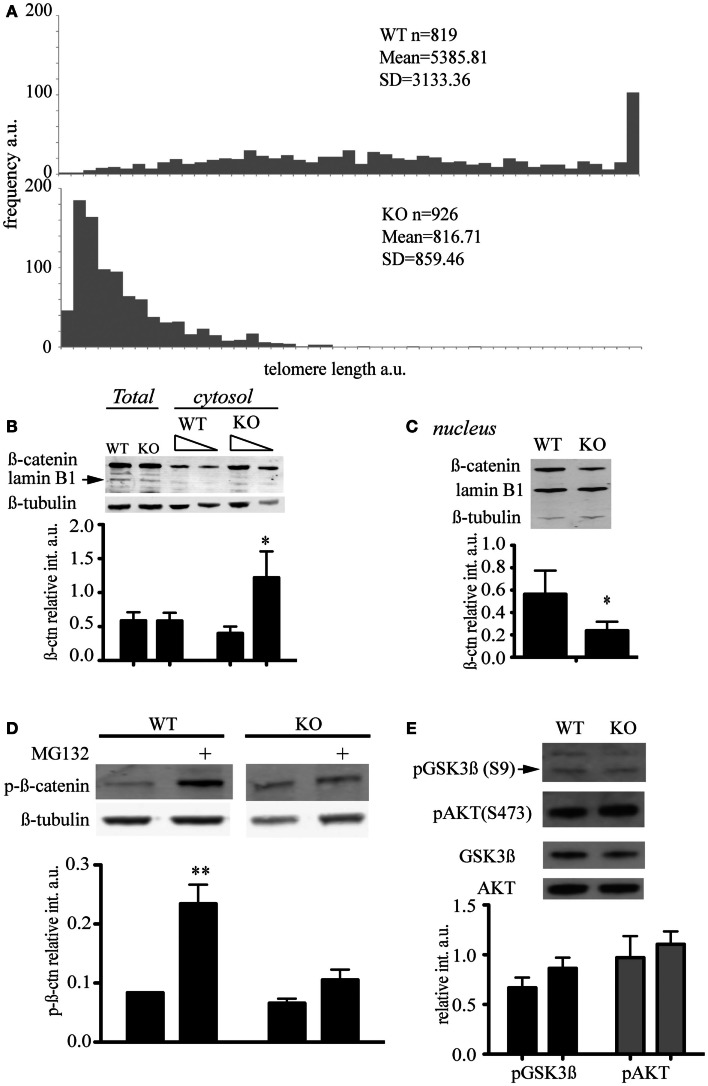
**Telomere shortening affects β-catenin cellular distribution and its degradation**. **(A)** Q-FISH analysis of wild-type (WT) and *mTert^−*/*−^* mESC (KO) at passage 61 and 70 respectively (*P* < 0.001). **(B)** Immunoblot of total and cytosolic cell extract of WT and KO mESC at passage>60. The membrane was probed with an anti-total β-catenin antibody and anti-β-tubulin antibody. Anti-lamin B antibody was used to assess the purity of the cytosolic fractionation (10 and 5 μg loaded, respectively). The signal for cytosolic β-catenin was quantified relative to the signal for β-tubulin (IRDye® Infrared Dyes, LI-COR Biosciences). **(C)** Immunoblot of nuclear cell extract of WT and KO mESC at a passage>60. The membrane was probed with an anti-total β-catenin antibody and an anti-lamin B1 antibody. Anti-β-tubulin antibody was used to assess the purity of the nuclear fractionation. The signal for nuclear β-catenin was quantified relative to the signal for lamin B1. **(D)** Immunoblot of phosphorylated β-catenin in cytosolic cell extracts treated with (+) and without the proteasome inhibitor MG132 (10 μM) for 6 h. The signal for cytosolic phospho-β-catenin was quantified relative to the signal for β-tubulin. The histograms in **(A–C)** represent the data of the blot shown plus two other independent data sets (average ± standard deviation; Student’s *t*-test: *P < 0.05, **P < 0.01). **(E)** Enhanced chemiluminescence immunoblot of total cell extract of WT and KO mESC probed with anti-phospho-GSK (S9), anti-total GSK, anti-phospho-AKT (S473), and anti-total AKT antibodies. The blot shown is representative of at least three independent experiments. The signal of p-GSK3β and p-AKT was quantified relative to the total unphosphorylated protein, GSK, and AKT respectively. The histograms represent the average of four independent analyses, *P* = 0.057 for p-GSK3β and *P* = 0.39 for p-AKT. Error bars indicate standard deviation.

Murine ESC lacking the telomerase reverse transcriptase were generated and characterized previously and show an accumulation of telomere signal-free ends at late passage (Liu et al., 2000; Erdmann et al., 2004). We queried whether the abundance of key signaling factors would be altered in the presence of short telomeres, and focused our investigation on β-catenin, a critical component of the Wnt signaling network that controls cell proliferation and differentiation (Clevers and Nusse, 2012). β-catenin distribution and post-translational modifications were compared in *mTert^−*/*−^* at late passage (>60 passages) and wild-type ESC at a similar passage number (Figure 2A). We observed that cytosolic β-catenin was significantly more abundant in *mTert^−*/*−^* ESC with critically short telomeres compared to wild-type cells (Figure 2B, Student’s *t*-test *P* = 0.003) while the total content remained unchanged (Figure 2B, *P* = 0.968). Accordingly, higher levels of nuclear β-catenin were observed in wild-type cells (Figure 2C, *P* = 0.027). Taken together, these results indicate that the distribution of β-catenin differed between the two cell types.

β-Catenin is a target of the GSK3β kinase which phosphorylates the residues S33/37, and T41. The tri-phosphorylated form of β-catenin is rapidly degraded by the proteasome (Liu et al., 2002). We used an antibody specific for the triple-phosphorylated β-catenin (S33/37, T41) to assess the phosphorylation status of β-catenin in ESCs with or without short telomeres, and found no difference in the levels of phosphorylated, cytosolic β-catenin (Figure 2E). Since the degradation of phospho-β-catenin occurs very rapidly and may mask subtle differences in abundance, we treated ESCs with the proteasome inhibitor MG132. In the presence of MG132, the difference in the phosphorylation status of cytosolic β-catenin in wild-type cells compared to *mTert^−*/*−^* ESC achieved statistical significance (Figure 2D, *P* = 0.0014). These results suggest that β-catenin is degraded less rapidly in *mTert^−*/*−^* ESC with short telomeres, or that there is a pool of β-catenin in cells with critically short telomeres that is immune to proteasome-dependent degradation.

As GSK3β activity is inhibited by the phosphorylation of a serine at amino acid position 9 (Sutherland et al., 1993; Desbois-Mouthon et al., 2001; Fukumoto et al., 2001), we assessed the serine 9 phosphorylation status of GSK3β. We did not detect a significant difference between wild-type and *mTert^−*/*−^* ESCs (Figure 2E). GSK3β is phosphorylated by the kinase AKT, whose activity is regulated by the phosphorylation of serine 473 (Alessi et al., 1997; Fukumoto et al., 2001). We did not observe a significant difference in the level of AKT phosphorylation between wild-type and *mTert^−*/*−^* cells (Figure 2E). These results suggest that downstream effectors of Wnt signaling remain unaltered in *mTert^−*/*−^* cells with critically short telomeres.

### Comparison of Wnt signal transduction

**Figure 3:**
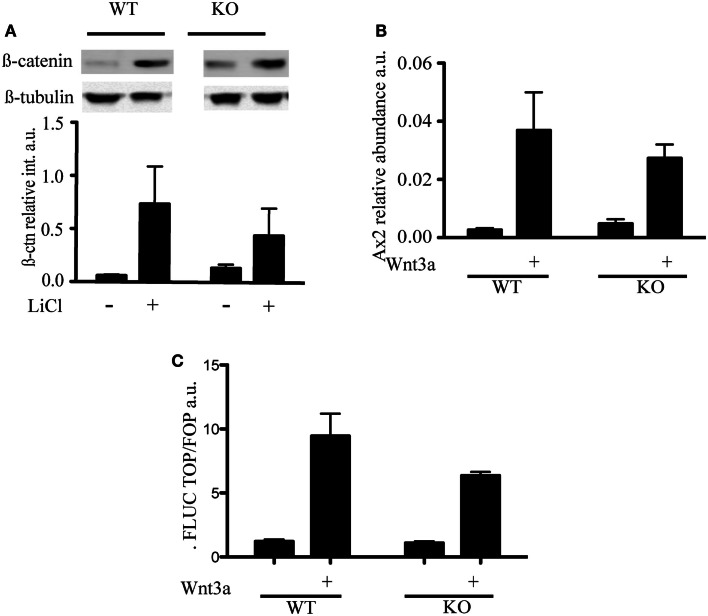
**Wnt signaling in telomerase knock-out mESC with short telomeres and β-catenin interactors**. **(A)** Immunoblot and quantification of β-catenin detected in cytosolic cell extracts of mESC treated with LiCl (30 mM) for 3 h. The intensity of β-catenin was normalized to the intensity of β-tubulin. The histogram represents the average (±standard deviation) of three independent experiments. **(B)** qRT-PCR to measure the levels of Axin2 mRNA transcript. WT and KO cells were treated with Wnt3a (100 ng/mL) for 3 days. The histogram represents the average (±standard deviation) of three independent qRT-PCR experiments, each performed with three replicates. **(C)** Transcriptional output of β-catenin activity measured using a TCF-driven luciferase reporter system. The histogram represents the average (±standard deviation) of the ratio of Renilla normalized firefly luciferase activity in Top versus Fop plasmid transfected cells of two independent experiments. Using a student’s *t*-test, no difference was noted in the presence or absence of Wnt3a (*P* = 0.223).

LiCl is an inhibitor of GSK3β that triggers phosphorylation on serine 9 through an as yet unknown mechanism (Rao et al., 2005a). Because inactivation of the kinase activity of GSK3β results in the inhibition of phosphorylation of β-catenin and its stabilization, LiCl treatment is often used to activate Wnt (Rao et al., 2005b). To assess whether the different distribution of β-catenin was associated with a difference in Wnt signaling, we treated mESC with LiCl and, as expected, observed a stabilization of β-catenin levels in both wild-type and *mTert^−*/*−^* ESCs (Figure 3A). We did not detect a significant difference in the transcription of a specific target of Wnt signaling, *Axin2*, in response to Wnt3a (Figure 3B). Similar to *Axin2*, a reporter system containing three consensus TCF binding sites upstream of the firefly luciferase gene did not exhibit a statistically significant difference between WT and KO cells (Figure 3C) (Korinek et al., 1998). Thus, two independent outputs of Wnt signaling were not appreciably altered in *mTert^−*/*−^* ESCs with short telomeres. The transcription of the cell cycle-regulated genes *c-myc* and *cyclinD1* are also regulated by Wnt and many other signaling networks, but did not exhibit a statistically significant trend in response to Wnt3a (Burdon et al., 2002; Jho et al., 2002) (data not shown).

In order to identify factors responsible for β-catenin cytosolic accumulation in *mTert^−*/*−^* ESCs with short telomeres, we compared the profile of β-catenin interacting proteins using mass spectrometry. Three independent β-catenin immunoprecipitations from total lysates were performed and only proteins recovered in all three experiments were considered (152 proteins in total). We considered a protein interaction significantly different between wild-type or *mTert^−*/*−^* ESC if it exhibited a peptide intensity ratio of<0.667 or>1.5 with an unpaired Student’s *t*-test *P* value < 0.05 (see Section Materials and Methods). Adenomatous polyposis coli (APC), was enriched by approximately twofold, (*P* = 0.03) in *mTert^−*/*−^* ESC relative to wild-type ESC (Table 1). APC interacts with β-catenin and together with Axin1, constitutes the scaffold of the destruction complex that regulates the stability of cytosolic β-catenin (Rubinfeld et al., 1993; Hart et al., 1998; Hamada and Bienz, 2004; Clevers and Nusse, 2012). Interestingly APC is also implicated in the regulation of the nuclear export of β-catenin and, therefore, influence the balance between nuclear and cytoplasmic β-catenin independently of Wnt signaling (Henderson, 2000). Eight other proteins were also identified as novel interactors of β-catenin and have not yet been further characterized (Table 1).

**Table 1:** **Results of IP-MS of β-catenin in WT and *mTert*^−*/*^^−^ mESC**.

Accession ID	Description	Total peptides	Peptides quantified	Average intensity WT a.u.	Average intensity KO a.u.	*P* value	Ratio KO/WT
Gi|86262157|ref|NP_808386.2|	Hypothetical protein LOC239796	4	3	0.00343	0.00155	0.00037	0.45091
Gi|124486588|ref|NP_001074475.1|	Sickle tail protein isoform c	28	28	0.05906	0.02017	0.00154	0.34160
Gi|40254129|ref|NP_258435.2|	Armadillo repeat protein deleted in velo-cardio-facial syndrome homolog	17	17	0.06524	0.05290	0.01504	0.81088
Gi|6755368|ref|NP_035426.1|	40S Ribosomal protein S18	5	5	0.01326	0.00710	0.01607	0.53595
Gi|31982755|ref|NP_035831.2|	Vimentin	11	8	0.00262	0.00749	0.01611	2.85760
Gi|31542151|ref|NP_038827.2|	Arginyl-tRNA-protein transferase 1 isoform 1	8	8	0.03619	0.06278	0.02867	1.73447
Gi|112807186|ref|NP_766307.2|	GCN1 general control of amino acid synthesis 1-like 1	2	2	0.00068	0.00027	0.02990	0.40295
Gi|110225370|ref|NP_031488.2|	Adenomatosus polyposis coli protein (APC)	35	35	0.05596	0.09804	0.03139	1.75192
Gi|79750409|ref|NP_075025.2|	Hamartin	7	7	0.00687	0.01120	0.03649	1.63012

*Nine proteins were differently represented in β-catenin immunoprecipitations of WT and *mTert*^−*/*−^ mESC. The stringency criteria used to determine hits are described in Section “Materials and Methods.” Note that β-catenin was immunoprecipitated in WT and *mTert^−*/*−^* cells with a comparable efficiency [over 20 peptides in all IPs and peptide intensity ratio (KO/WT) of 0.97]*.

### Rescue of cytosolic β-catenin with telomere lengthening

**Figure 4:**
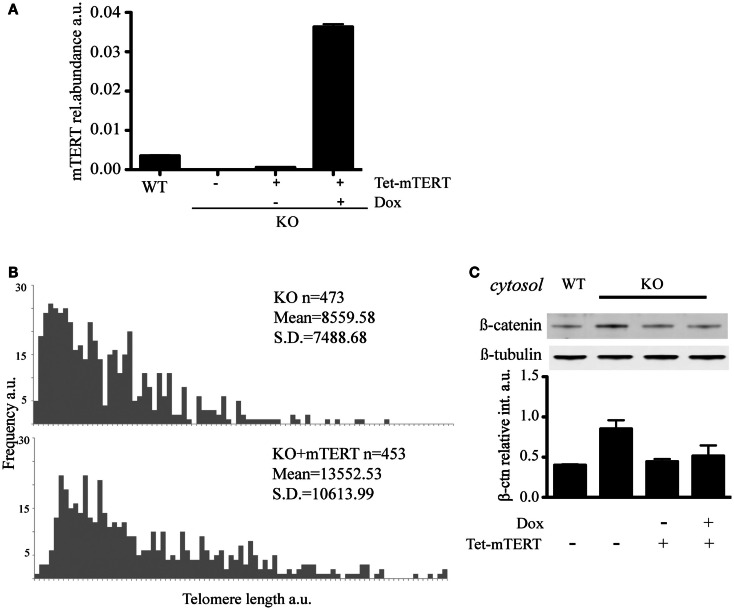
**(A)** qRT-PCR to quantify mTert transcript in WT, KO, and KO cells re-transfected with inducible mTert under a Doxycyclin inducible promoter (see text for details). The histogram represents the average (±standard deviation) of three qPCR replicates. **(B)** Q-FISH to evaluate telomere lengthening in KO cells re-transfected with mTERT under the induction of Doxycyclin for 70 days. **(C)** Rescue of the level of cytosolic β-catenin in KO cells stably re-transfected with mTert under the control of an inducible promoter. Doxycycline (Dox) was used to induce *mTert* transcription. The results were verified with two independent protein extractions.

To address whether the reintroduction of telomerase and extension of telomeres could restore the level of cytosolic β-catenin, we reintroduced *mTert* into *mTert^−*/*−^* ESCs under the control of a tetracycline-inducible promoter and, after selection of *mTert*-positive clones, cells were propagated under *mTert* induction conditions (+Dox) for 70 days (Figures 4A,B). The reactivation of telomerase upon addition of doxycycline was confirmed by TRAP (telomerase repeat amplification protocol, data not shown) and the extension of the telomeres verified by Q-FISH analysis (Figure 4B). At this point, the culture was split in two and propagated in the absence (−Dox) or presence (+Dox) of *mTert* for an additional four population doublings (Figures 4A,B). Analysis of the level of cytosolic β-catenin in ESCs with extended telomeres, irrespective of *mTert* expression, revealed a rescue of the cytosolic β-catenin to levels comparable to wild-type ESCs (Figure 4C, *P* = 0.20). This result suggests that the distribution of β-catenin is dependent on telomere length rather than telomerase activity. This result is similar to the finding that mice or ESCs lacking telomerase activity do not exhibit phenotypes until telomeres become critically shortened (Erdmann and Harrington, 2009; Strong et al., 2011). Instead, a loss of tissue self-renewal is evident at generations above G4, underscoring the dependence of the phenotype upon loss of telomere integrity (Vidal-Cardenas and Greider, 2010; Strong et al., 2011).

## Discussion

Here, we discussed the impact of telomere integrity on cell signaling. We show new data that mESC with short telomeres undergo an accumulation of cytosolic β-catenin. Although the level of nuclear β-catenin is higher in wild-type cells, this difference does not result in an induction in the transcription of Wnt target genes. This observation is in general agreement with the finding that activation of Wnt signaling leads to β-catenin nuclear import, but there is no relationship between the level of nuclear β-catenin and Wnt activation (Guger and Gumbiner, 2000). The higher cytosolic content of β-catenin in *mTert^−*/*−^* ESCs might be the result of an altered balance of β-catenin nuclear import/export or of β-catenin degradation/stabilization. In support of the first explanation, we observed an enrichment of APC in β-catenin immunoprecipitates from *mTert^−*/*−^* cells. APC shuttles between the nucleus and the cytoplasm independently from other factors of the destruction complex (Henderson, 2000). The destruction complex is not disassembled in the presence of Wnt; instead, degradation of β-catenin by the proteasome is altered upon Wnt stimulation (Hilger and Mann, 2012; Li et al., 2012). This finding may explain why an increased level of APC-β-catenin complex might not necessarily result in higher β-catenin degradation. Furthermore, our finding that the phosphorylation of β-catenin is increased in wild-type ESCs but not *mTert^−*/*−^* ESCs in the presence of the proteasome inhibitor MG132 supports the notion that the activity of the proteasome is altered in *mTert^−*/*−^* ESCs with critically short telomeres. Such an effect on the proteasome may not be surprising as previous studies have demonstrated a link between cellular aging and an alteration of the ubiquitin-proteasome machinery (Grillari et al., 2006). These results support the notion that the up-regulation of β-catenin occurs as result of altered protein degradation in the presence of short telomeres. Further analysis of the complex composition, stoichiometry, and relative abundance of these complexes in the nucleus and cytoplasm in response to critically short telomeres will be informative.

It remains to be tested whether difference in β-catenin phosphorylation status in murine ESCs with critically short telomeres also impacts Wnt signaling more generally. β-catenin intersects several signaling cascades, not all of which are linked to Wnt (Valenta et al., 2012). For example, in the presence of LIF, β-catenin transcriptional activity is not required to maintain self-renewal; it is mostly its role at the cell adhesion structures that is required to allow differentiation (Lyashenko et al., 2011). On the cytoplasmic side of the plasma membrane, β-catenin interacts with cadherins and α-catenin to stabilize cell–cell adhesion structures but also to regulate cytoskeleton dynamics (Yamada et al., 2005). The majority of these interactions with its partners are regulated by phosphorylations at sites other than the S33, 37, T41 (Liu, 1999). In general, the pattern of β-catenin phosphorylation regulates the transition from a structural versus signaling role (Valenta et al., 2012). Thus, the impact of critically short telomeres upon the phophorylation of β-catenin at sites other than S33, 37, T41 should also be investigated.

**Figure 5:**
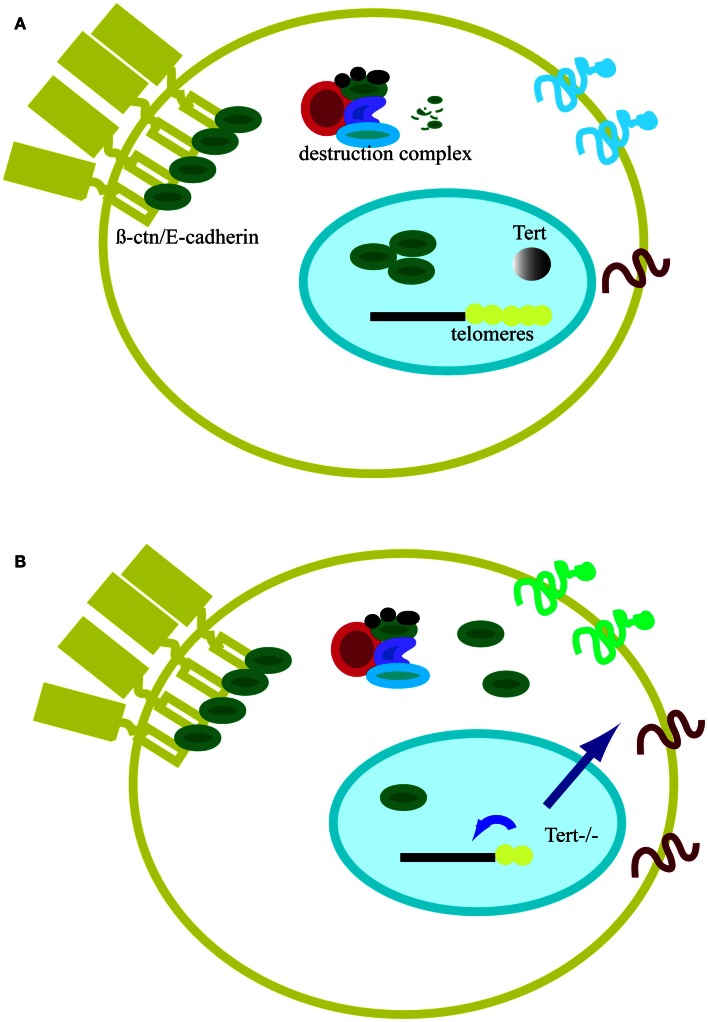
**Model of telomeres as platform for cell signaling**. **(A)** In the presence of telomerase, telomeres remain stable. In the absence of Wnt, cytosolic β-catenin (β-ctn, green particle) associates with the APC (red particle) and the kinase GSK3β (blue particle). Free β-catenin is rapidly degraded by the proteasome. The majority of β-catenin is bound to the cell membrane in a complex with E-cadherin (yellow element at the plasma membrane). **(B)** In the absence of telomerase, telomeres shorten and the protective effect of shelterin is eventually lost (yellow dots). Telomere shortening affects epigenetics marks in sub-telomeric regions and at the global genome level (small blue arrow). Signals emanating from telomeres, directly or indirectly, can alter cell signaling. One sign of this influence is the accumulation of β-catenin in the cytosol as result of proteasome impairment or higher nuclear export by APC. Cell signaling changes may also affect proteins expressed at the cell membrane and influence cell adhesion properties (larger blue arrow).

In conclusion, global genomic changes driven by short telomeres have consequences on general gene expression and cell metabolism (Figures 5A,B). For example, critically short telomeres in mESCs lacking *Tert* influence DNA and histone methylation at the promoters of pluripotency regulators such as *Nanog* and *Oct4*, thereby negatively affecting the stable differentiation of mESCs (Pucci et al., 2013). In the nuclear compartment, β-catenin interacts with several chromatin remodeling complexes, including the histone acetylase p300/CBP and the helicases TIP49a/Pontin52 and TIP49b/Pontin52 and Brg1, the latter of which also interacts with telomerase (Mosimann et al., 2009; Park et al., 2009). β-catenin increases H3K4 methylation at the *c-myc* promoter through its interaction with the histone methyltransferase SET1. Interestingly, this activity is counteracted by Apc, which displaces β-catenin from the chromatin remodeling complex (Sierra et al., 2006). Furthermore, the involvement of β-catenin in the control of telomerase transcription and the previous finding of complexes containing both telomerase and β-catenin at promoter sequences show that β-catenin and telomere integrity are connected (Park et al., 2009; Hoffmeyer et al., 2012). Hence, it is reasonable to postulate that the alteration of binding of telomeric proteins or epigenetic modifications can trigger signaling cascades that might culminate in changes at the plasma membrane and alter communication with the environment (Figures 5A,B). Although the precise means by which short telomeres elicit genome-wide changes in gene expression is unknown, one candidate mechanism is RAP1, a transcription factor that binds extra-telomeric sites and in whose absence there are a number of changes in gene expression in processes related to cell metabolism, cell adhesion, and cancer (Martinez et al., 2010; Martinez and Blasco, 2011). Moreover, together with *Trf2*, *Rap1* transcription is directly regulated by β-catenin (Diala et al., 2013). Taken together, these findings reinforce the notion that β-catenin and telomere structure and function are interconnected. Clearly, the future promises to uncover additional intriguing links between the impact of critically short telomeres and cytoplasmic cell signaling.

## Materials and Methods

### Cell culture

Wild-type and telomerase reverse transcriptase-deficient mESC (E14) were cultured in GMEM, 15% v/v FBS (Hyclone, UK), β-mercaptoethanol, penicillin/streptomycin, and leukemia inhibitory factor (Sigma, UK), and split by a ratio of 1:8 every 3 days, as described in Erdmann et al. (2004). The absence or presence of telomerase activity was assessed by the TRAP (telomerase repeat amplification protocol), performed following manufacturer’s instructions (TRAPeze, Millipore, UK). Cells grown in 6-well plates were lysed in 50 μL of CHAPS 1 × buffer. Two microliters of cell lysate were assayed in the TRAP.

### Cell fractionation and immunoblotting

Cells grown in 10-cm diameter plates were washed and scraped in PBS. For cell fractionation, cells were pelleted for 5 min at 1500 × *g* and re-suspended in hypotonic lysis buffer (50 mM Tris, pH 7.8, 250 mM sucrose, 2 mM EDTA) supplemented with Roche’s Complete Protease Inhibitor Cocktail and PhoSTOP Phosphatase Inhibitor Cocktail. Cells were homogenized with 20 strokes in a Dounce homogenizer and then centrifuged for 10 min at 2800 × *g* to precipitate the nuclei. The supernatant represented the cytosolic fraction. The nuclear pellet was re-suspended in buffer S1 (0.25 M sucrose, 10 mM MgCl_2_), layered over an equal volume of buffer S3 (0.88 M sucrose, 0.5 M MgCl_2_), and centrifuged at 2800 × *g* for 10 min. The pellet was re-suspended in RIPA buffer (50 mM Tris, pH 7.5, 150 mM NaCl, 1% v/v NP-40, 0.5% w/v deoxycholic acid) containing protease and phosphatase inhibitors. The nuclear extract was sonicated and centrifuged at maximum speed 16,000 × *g* for 10 min to obtain the nuclear extract. The protein concentrations of the cytosolic and nuclear lysates were measured using the Bradford method prior to loading onto gels for SDS-PAGE. In all experiments, 10 μg of cytosolic protein and 2 μg of nuclear protein were loaded. For total cell extracts, cells were scraped and re-suspended in RIPA buffer. Ten micrograms of total protein lysate, measured by the Bradford method, were loaded onto gels for SDS-PAGE. SDS-PAGE was performed with NuPAGE Bis–Tris 4–12% w/v gradient gels (Invitrogen, UK). After electrophoresis, the proteins were transferred onto an Immobilon-FL membrane (Millipore) at a constant 100 V for 1 h. The membrane was blocked in 5% w/v skimmed milk powder (non-fat) in TBST. Primary antibodies were incubated with the membrane overnight at 4 °C in 2.5% w/v skimmed milk powder in TBST. The primary antibodies used were as follows: rabbit polyclonal anti-β-catenin (1:4000; Bethyl Laboratories, Inc., USA); rabbit monoclonal anti-β-catenin, clone E247 (1:4000; Millipore); rabbit polyclonal anti-phospho-β-catenin (Ser33/37/Thr41) (1:2000; Cell Signaling UK); anti-E-cadherin (1:4000; BD Biosciences UK); rabbit anti-phospho-GSK3β (S9) and mouse anti-GSK3β total (both at 1:1000; Cell Signaling); rabbit anti-phospho-AKT (S473) and rabbit anti-AKT total (both at 1:2000; Cell Signaling). Mouse anti-β-tubulin (1:4000; Sigma) was used as a loading control for the total protein extracts and cytosolic fractions, whereas rabbit anti-lamin B1 (1:2000; a gift from Dr. Eric Schirmer) was used for nuclear fractions. Secondary antibodies were HRP-conjugated anti-mouse and anti-rabbit (1:10000 and 1:5000, respectively; GE Healthcare). For quantification of the immunoblot bands, secondary antibodies were donkey anti-mouse (IRDye 800) and donkey anti-rabbit (IRDye 680) (1:10000 and 1:5000, respectively; LI-COR Biosciences, UK). For Wnt3a treatment, 100 ng/mL of recombinant mouse Wnt3a (Millipore) was added to the culture medium for 3 days. For LiCl treatment, 30 mM LiCl was added to the culture medium for 4 h. For the inhibition of the proteasome, cells were treated with 10 μM of MG132 (Sigma, UK, dissolved in DMSO) for 6 h. Cells were collected by scraping and lysed as previously described for cell fractionation and western blot analysis.

### Western blot quantitative analysis

Images of membranes probed with secondary IRDye antibodies were acquired with an Odyssey scanner and analyzed with Odyssey software (Licor Biosciences). Excel and GraphPad Prism v.5 were used for statistical analysis. Briefly, two rectangles of the same size were placed over β-catenin and the relevant control (β-tubulin or lamin B1 for cytosol or nuclear extract, respectively). The intensity of β-catenin was normalized to the value of the loading control within the same lane and averaged against at least three independent replicates. The Student’s *t*-test was used to evaluate the statistical significance of the comparison (Gardano et al., 2011).

### Quantitative fluorescence *in situ* hybridization

The Q-FISH protocol was carried out as described (Liu et al., 2000). Metaphase spreads were captured using Metafer 4 software and analyzed using Isis software. Statistical analysis of telomere intensity distribution was performed using Welch’s unpaired *t*-test.

### qRT-PCR

RNA was extracted from cells grown in 6-well plates using Qiagen’s RNeasy Mini Kit. The RNA was treated with DNase for 1 h prior to the reverse transcription reaction. One microgram of RNA was retrotranscribed with random primers (Invitrogen) using SMART MMLV reverse transcriptase (Clontech Laboratories, Inc., USA). The cDNA mixture was diluted 20 times in water containing RNase before proceeding with the qPCR (Lightcycler 480, Roche, UK). The sequences of the primers used were as follows: Axin2 forward 5′-AGCGCCAACGACAGCGAGTT-3′; Axin2 reverse 5′-TCCCCATGCGGTAAGGAGGGAC-3′; GAPDH forward 5′-AGGTCGGTGTGAACGGATTTG-3′; GAPDH reverse 5′-TGTAGACCATGTAGTTGAGGTCA-3′mTERT forward 5′-TTCTAGACTTGCAGGTGAACAGCC-3′; mTERT reverse 5′-TTCCTAACACGCTGGTCAAAGGGA-3′. Data were analyzed with Excel and GraphPad Prism v.5.

### Top-flash experiments

Cells were seeded at a concentration of 2.5 × 10^4^ mL in 12-well dishes and, 24 h later, Extreme Gene 9 (Roche, UK) was used to transfect 0.5 μg DNA (in total) consisting of Top-firefly luciferase plasmid or the negative control Fop-firefly luciferase (Millipore) and 0.05 μg of Renilla plasmid transcribed with a SV40 promoter, pRL (Promega). Cell lysis was performed 48 h after transfection with the Passive Lysis buffer supplied by the Dual luciferase assay (Promega, UK). Firefly and Renilla luciferase activities were monitored following manufacturer’s instructions. Luciferase activity was recorded using an Infinite 200 instrument (Tecan group Ltd.). Wnt3a treatment was performed as previously described. Top-firefly luciferase signals were normalized to renilla luciferase values and then normalized to Fop-luciferase activity for each respective treatment, i.e., with or without Wnt3a. Graphpad Prism v.5 was used for statistical analysis.

### mTERT cell transfection and plasmids

The plasmid pTRE-Bi-*Tert*-IRES-EGFP-Hygro was constructed by amplification of *Tert* cDNA by PCR and insertion into pTRE-Tight-Bi (Clontech) following digestion with *Eco*RI and *Sal*I. IRES-EGFP sequence was obtained from pCAGMKOSiE (kindly provided by K. Kaji) and inserted into pTRE-Tight-Bi (following digestion with *Sal*I and *Eco*RV) using *Sal*I and *Hpa*I sites and then inserted into pTRE-Bi-*Tert* using *Not*I sites. Finally, the hygromycin resistance gene was cloned by PCR into the *Xba*I restriction site of pTRE-Tight-Bi and pTRE-Bi-*Tert*-IRES-EGFP vectors to create pTRE-Bi-EGFP-Hygro and pTRE-Bi-*Tert*-IRES-EGFPHygro. The pCAG-rtTA-advanced vector was constructed by removal of the MKOS ORFs from CAGMKOSiE with *Eco*RI and *Bam*HI and replacement with the advanced tetracycline reverse transactivator sequence (Clontech) (Pucci et al., 2013).

### Immunoprecipitation and mass spectrometry

Wild-type and *mTert^−*/*−^* ESC were propagated in three 15-cm diameter plates for each immunoprecipitation. Three independent immunoprecipitations were performed contemporaneously on wild-type and *mTert^−*/*−^* ESC. Cells were lysed in 1 mL of lysis buffer (50 mM Tris, pH 7.5, 5 mM EDTA, 5 mM NaF, 10% v/v glycerol, 0.1% v/v NP-40, 1 mM DTT) supplemented with Roche’s Complete Protease Inhibitor Cocktail. Following centrifugation at 16,000 × *g* for 10 min, the amount of total protein in all the samples was assessed by the Bradford method. Ten micrograms of rabbit monoclonal anti-β-catenin antibody (clone E247, Millipore) were added to each lysate and incubated for 2 h at 4 °C with rocking. Magnetic Dynabeads Protein A (Invitrogen) was equilibrated in the lysis buffer prior to addition to cell lysates (10 μL beads added to each immunoprecipitation). The bead/lysate mixtures were then incubated for 40 min at 4 °C. Following four washes with a washing buffer (lysis buffer without glycerol), the beads were re-suspended in 20 μL washing buffer. The samples were boiled in Laemmli buffer and loaded onto a NuPAGE Bis–Tris 4–12% v/v gradient gel for SDS-PAGE. The gel was stained with SimplyBlue SafeStain (Life Technologies, UK). Each entire gel lane was sliced into six pieces, then processed according to an in-gel protocol for trypsin digestion.

Capillary-HPLC-MS/MS analysis was performed on an on-line system consisting of a micro-pump (1200 binary HPLC system, Agilent, UK) coupled to a hybrid LTQ-Orbitrap XL instrument (Thermo-Fisher, UK). MS/MS data was searched using MASCOT (Matrix Science Ltd, UK) against the *Mus musculus* subset of the NCBI protein database using a maximum missed-cut value of 2. Variable methionine oxidation, ST and Y phosphorylation, and N-term acetylation were used and fixed cysteine carbamidomethylation were used in all searches; precursor mass tolerance was set to 7 ppm and MS/MS tolerance to 0.4 amu. The significance threshold (*p*) was set below 0.05 (MudPIT scoring). A peptide Mascot score cut-off of 20 was used in the final analysis, which corresponds to a global false discovery rate of 3.6% using a decoy database search. LC-MS label-free quantification was performed using Progenesis (Non-linear Dynamics, UK). For label-free quantitation, the total number of Features (i.e., intensity signal at a given retention time and m/z) was reduced to MS/MS peaks with charge of 2, 3, or 4+ and only the five most intense MS/MS spectra were retained per “Feature.” The subset of multicharged ions (2+, 3+, 4+) was extracted from each LC-MS run. Protein quantification was performed as follows; for each protein, the associated unique peptide ions were summed to generate an abundance value and normalized by dividing the protein intensity by the bait intensity (β-catenin). The within group means were calculated to determine the fold change and a *t*-test was used between the two groups. Regarding quantitative cut-off thresholds, proteins were considered a hit if two or more peptides were detected with an absolute ratio of at least 1.5 (i.e., 1.5 fold increase, or 0.667 decrease) and a significance of *p* < 0.05. Nine proteins met this threshold criteria (Table 1).

## Conflict of Interest Statement

The authors declare that the research was conducted in the absence of any commercial or financial relationships that could be construed as a potential conflict of interest.
